# Management of insecticides for use in disease vector control: a global survey

**DOI:** 10.1186/s12879-021-06155-y

**Published:** 2021-05-22

**Authors:** Henk van den Berg, Haroldo Sergio da Silva Bezerra, Emmanuel Chanda, Samira Al-Eryani, Bhupender N. Nagpal, Elkhan Gasimov, Raman Velayudhan, Rajpal S. Yadav

**Affiliations:** 1 Laboratory of Entomology, Wageningen University, PO Box 16, 6700AA Wageningen, The Netherlands; 2 Department of Communicable Diseases and Environmental Determinants of Health, Pan-American Health Organization/World Health Organization, Washington, DC USA; 3 WHO Regional Office for Africa, Brazzaville, Congo; 4 WHO Regional Office for the Eastern Mediterranean, Cairo, Egypt; 5 WHO Regional Office for South-East Asia, New Delhi, India; 6 WHO Regional Office for Europe, Copenhagen, Denmark; 7 Department of Control of Neglected Tropical Diseases, World Health Organization, 20 Avenue Appia, 1211, 27 Geneva, Switzerland

**Keywords:** Insecticide resistance monitoring, Insecticide management, Pesticide management, Safety precautions, Vector control operations

## Abstract

**Background:**

Vector control plays a critical role in the prevention, control and elimination of vector-borne diseases, and interventions of vector control continue to depend largely on the action of chemical insecticides. A global survey was conducted on the management practices of vector control insecticides at country level to identify gaps to inform future strategies on pesticide management, seeking to improve efficacy of interventions and reduce the side-effects of chemicals used on health and the environment.

**Methods:**

A survey by questionnaire on the management practices of vector control insecticides was disseminated among all WHO Member States. Data were analysed using descriptive statistics in MS Excel.

**Results:**

Responses were received from 94 countries, or a 48% response rate. Capacity for insecticide resistance monitoring was established in 68–80% of the countries in most regions, often with external support; however, this capacity was largely lacking from the European & Others Region (i.e. Western & Eastern Europe, North America, Australia and New Zealand). Procurement of vector control insecticides was in 50–75% of countries taking place by agencies other than the central-level procuring agency, over which the central authorities lacked control, for example, to select the product or assure its quality, highlighting the importance of post-market monitoring. Moreover, some countries experienced problems with estimating the correct amounts for procurement, especially for emergency purposes. Large fractions (29–78%) of countries across regions showed shortcomings in worker safety, pesticide storage practices and pesticide waste disposal. Shortcomings were most pronounced in countries of the European & Others Region, which has long been relatively free from mosquito-borne diseases but has recently faced challenges of re-emerging vector-borne diseases.

**Conclusions:**

Critical shortcomings in the management of vector control insecticides are common in countries across regions, with risks of adverse pesticide effects on health and the environment. Advocacy and resource mobilization are needed at regional and country levels to address these challenges.

**Supplementary Information:**

The online version contains supplementary material available at 10.1186/s12879-021-06155-y.

## Background

Human diseases transmitted by arthropod vectors account for approximately 17% of the estimated global burden of infectious diseases [1]. The most serious vector-borne diseases in terms of disease burden are malaria, dengue, lymphatic filariasis, onchocerciasis and leishmaniasis [2]. In many tropical and subtropical regions around the world, human populations are at risk from multiple vector-borne diseases [3]. The critical importance of vector control in managing these diseases has been emphasized [2, 4, 5]. For some diseases, such as dengue, zika and chikungunya, vector control and interruption of human-vector contact are the only available control options [6]; for other diseases, such as malaria, vector control plays a major role in control and elimination efforts [7].

In 2017, the World Health Assembly adopted the Global Vector Control Response (GVCR) as a strategy to strengthen capacity and coordination for vector control and public health entomology [1]. The GVCR is aligned with the key elements of the integrated vector management (IVM) approach, which seeks to make vector control more effective, efficient and sustainable, through evidence-based decision-making, intersectoral collaboration and an integrated approach to implementation [8, 9].

For the implementation of vector control, the GVCR calls for the use of efficacious interventions, with availability of high-quality vector control products with capacity for optimal application while minimizing the risks of pesticides to health and the environment. The mainstay methods for vector control depend largely on the action of chemical pesticides (specifically, insecticides), applied on substrates, on water surfaces, as spatial sprays or impregnated in netting materials. Hence, appropriate management practices are required throughout the pesticide lifecycle, including on pesticide procurement, transport, storage, application and disposal [10]. Moreover, the routine monitoring and management of insecticide resistance is vital to preserve susceptibility to available vector control products in vector populations [11]. Not having insecticide resistance management in place can cause diseases to resurge [12].

This paper describes the results of a global assessment on the management practices of vector control insecticides at country level. The objective is to identify gaps to inform future strategies on pesticide management, seeking to improve efficacy of vector control interventions and reduce the adverse effects of pesticides on health and the environment.

## Methods

The study was part of a comprehensive assessment of the global situation of agricultural pesticides and public health pesticides; the results of the comprehensive assessment have been documented in a different form as a separate report [13]. A questionnaire was prepared on the use and application of pesticides for vector-borne disease control, including procurement, insecticide resistance monitoring, quality control, safety precautions, storage, waste disposal and institutional aspects (see Additional File 1). Other components of the pesticide lifecycle, including manufacture, trade and regulatory control, are dealt with in a separate contribution [14]. The evaluation of operational procedures was not included in the questionnaire. Other public health pesticides, such as those directly applied on humans, household pest control products and professional public health pest control products, were not the focus of the questionnaire.

The questionnaire, together with a document explaining the purpose and use of the survey, was translated from English into French and Spanish and disseminated through e-mail as editable Word documents from WHO’s headquarters via its regional and country offices to the national focal point in the Ministry of Health in each country. All 194 Member States of WHO were targeted for the survey. The questionnaire was distributed in December 2017. At country level, the national focal point was requested to have the questionnaire completed by the director of the main national vector-borne disease control programme (e.g. malaria, dengue), or (where applicable) by the national manager for vector control (i.e. the person who has overall responsibility for entomological surveillance and vector control in the country). In countries with more than one national programme for vector-borne disease control, the malaria programme or vector control manager was requested to coordinate completion of the questionnaire.

For analysis of results, countries were grouped according to the United Nations Regional Groups of Member States, referred to as regions [15]. This classification differentiates the African, Asia-Pacific, Latin American & Caribbean, Eastern European, and Western European & Others groups of countries. It is noted that the Western European & Others Group includes Australia, Canada, New Zealand and the United States of America in addition to Western European countries. Because there were only four responses from the Eastern European Group, the data of the Eastern European Group were pooled together with those of the Western European & Others Group into the ‘European & Others Group’.

Data were analysed using descriptive statistics using MS Excel. Questions with binary responses were selected for analysis. Questions with narrative responses, questions that appeared to be ambiguous in retrospect, and several questions that had been used in a more broad-based pesticide study [14] were excluded from the analysis.

## Results

**Fig. 1:**
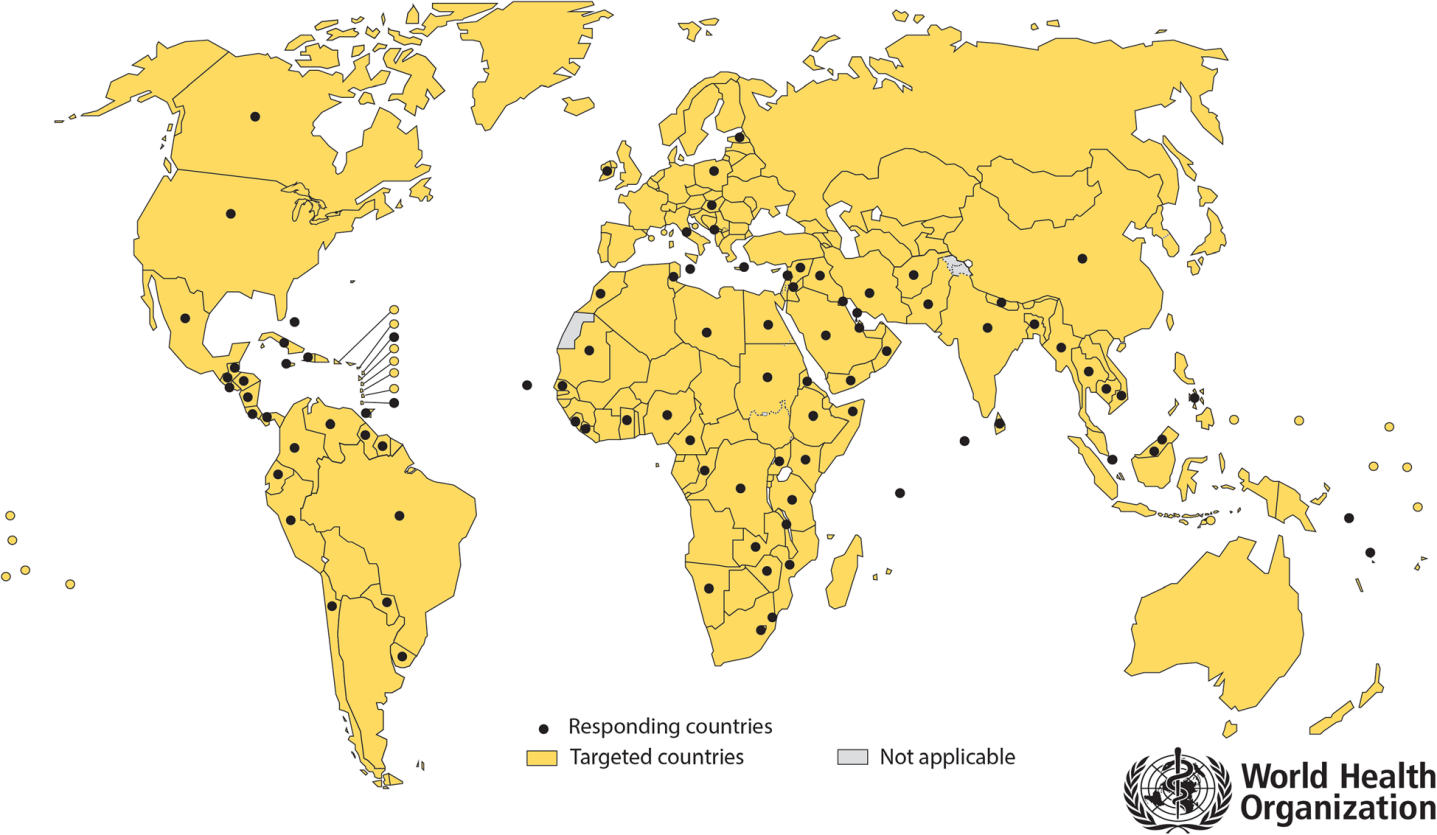
Map showing targeted and responding countries. Data source: World Health Organization (WHO). Map production: Control of Neglected Tropical Diseases, WHO. Written permission to use and adapt the map was granted by WHO. The boundaries and names shown, and the designations used on this map do not imply the expression of any opinion whatsoever on the part of the World Health Organization concerning the legal status of any country, territory, city, or area or of its authorities, or concerning the delimitation of its frontiers or boundaries. Dotted lines on maps represent approximate border lines for which there may not yet be full agreement. © WHO 2020. All rights reserved

By December 2018, questionnaire responses had been received from 94 out of 194 targeted countries, indicating a response rate of 48% (Fig. 1). The response rate was 29 out of 54 targeted countries (54%) from the African; 30 out of 55 (55%) from the Asia-Pacific; 25 out of 33 (76%) from the Latin American & Caribbean; and 10 out of 52 (19%) from the European & Others regions. Large countries were more likely to respond than small countries. Hence, when weighted for population per country, using population data for 2019 [16], the response rate was 73% for the African, 88% for the Asia-Pacific, 90% for the Latin American & Caribbean, and 38% for the European & Others regions.

Capacity for insecticide susceptibility testing (i.e. WHO or United States Centres for Disease Control and Prevention (CDC) phenotypic bioassays) was reportedly in place in most countries (68–80%) across regions (Table 1). An exception was the European & Others Region where the capacity for testing was less common (30% of responding countries), a result which is in line with the region’s low incidence of vector-borne diseases in recent history. Representative sentinel sites, needed for monitoring of temporal changes in the prevalence of resistance, had been established in 36–57% of the countries in the Africa, Asia-Pacific and Latin American & Caribbean regions (Table 1). Insectaries to support insecticide susceptibility testing and efficacy testing were reported to be in place in 40–68% of countries across regions. Capacity for molecular testing and biochemical testing was reported from few countries but was most common in the African Region (36–39% of countries) (Table 1). Out of the 25 countries that reported the presence of molecular testing capacity, 20 also reported the presence of biochemical testing capacity. In total, 24 out of 25 countries with molecular testing and 23 out of 24 countries with biochemical testing also reported that capacity for susceptibility testing was in place. This suggests that molecular and biochemical testing were not used on their own, but together, and in combination with susceptibility testing. Furthermore, out of a total of 93 responding countries, 14 reported having all components (i.e. susceptibility testing, sentinel sites, insectaries, molecular and biochemical testing) in place.

**Table 1:** Capacity for insecticide resistance monitoring

	United Nations Regional Group							
	African		Asia-Pacific		Latin American & Caribbean		European & Others^a^	
Topic	%	(n)	%	(n)	%	(n)	%	(n)
Insecticide susceptibility testing	68	(28)	70	(30)	80	(25)	30	(10)
Representative sentinel sites established	50	(28)	57	(30)	36	(25)	10	(10)
Insectaries in place for bioassays	57	(28)	50	(30)	68	(25)	40	(10)
Molecular testing of resistance	39	(28)	17	(30)	28	(25)	20	(10)
Biochemical testing of resistance	36	(28)	20	(30)	24	(25)	20	(10)

Data presented as % of responding countries per Regional Group that gave a positive response regarding each topic (n indicates number of responding countries for each topic)

^a^ Western & Eastern Europe, North America, Australia and New Zealand

Pesticide procurement is a demanding process to ensure the availability of correct amounts of quality-assured products that are efficacious against targeted vectors. Some 80–92% of all countries in the Asia Pacific, African and Latin American & Caribbean regions claimed that insecticide susceptibility was factored into the procurement process (Table 2). Despite this, a smaller percentage (68–80%) had capacity for insecticide susceptibility testing in place (see Table 1). Out of a total of 75 countries that used insecticide susceptibility status as criterion in the procurement process, 17 countries did not have susceptibility testing capacity. This suggests that the procurement requirements could not be fulfilled everywhere, unless some countries sent entomological samples for testing abroad.

**Table 2:** Conditions and challenges of procurement of vector control insecticides

	United Nations Regional Group							
	African		Asia-Pacific		Latin American & Caribbean		European & Others^a^	
Topic	%	(n)	%	(n)	%	(n)	%	(n)
Insecticide susceptibility status as criterion for selection	89	(28)	80	(30)	92	(25)	38	(8)
Problems estimating amounts needed for routine/normal situations	15	(27)	27	(30)	8	(24)	13	(8)
Problems estimating amounts needed for emergency situations	32	(28)	40	(30)	17	(24)	11	(9)
Quality control (pre- and/or post-shipment) required for procurement	56	(27)	73	(30)	36	(25)	0	(9)
Procurement requirements aligned with other countries	22	(27)	32	(28)	52	(25)	20	(10)

Data presented as % of responding countries per Regional Group that gave a positive response regarding each topic (n indicates number of responding countries for each topic)

^a^ Western & Eastern Europe, North America, Australia and New Zealand

A small fraction of countries (8–27%) reported that problems were encountered with estimating the appropriate amounts of vector control insecticides to be procured for normal or routine situations (Table 2). However, a substantially larger fraction of countries (11–40%) experienced problems estimating the amounts needed for emergency situations (e.g. disease outbreaks), particularly in the African and Asia-Pacific regions.

In 73% of Asia-Pacific countries, a requirement for procurement of vector control insecticides was that quality control was conducted before and/or after shipment into the country (Table 2). This requirement was less common in the other regions (0–56% of countries), suggesting that in many countries the quality of procured consignments was not guaranteed. Pesticide procurement may benefit from regional collaboration, for example, by combining the procurements of minor-use products between neighbouring countries to reduce costs. In this regard, 52% of countries in the Latin American & Caribbean Region reported that procedures, requirements and guidelines for procurement were aligned with those of other countries in the (sub-) region, whereas such alignment was less common in other regions (20–32%) (Table 2).

Most countries (63–84%), except in the European & Others Region, reported that the Ministry of Health procured pesticides for malaria control at central level (Table 3). Fewer countries in the African and Asia-Pacific regions reported central-level pesticide procurement for arboviral diseases (23–57%) and other vector-borne diseases (41–55%) (Table 3). The presence of central-level procurement does not mean that all vector control insecticides were procured that way. In 50–75% of countries there were other agencies or authorities apart from the central-level procuring agency, that procured pesticides for vector control (Table 3). These agencies or authorities, as reported by 69 countries, were local authorities, the private sector, donor-funded projects, and ministries other than health (Table 4). In the African Region, the private sector and donor-funded projects (e.g. the U.S. President’s Malaria Initiative) were the most commonly reported procuring agencies apart from the Ministry of Health. Local authorities were the most common procuring agencies besides the central Ministry of Health in the Asia-Pacific and Latin American & Caribbean regions, reported from 48 and 67% of countries, respectively (Table 4). In 22% of responses, procurement was centralized only; in 20% of responses, procurement was decentralized only; and in 58% of responses, procurement was both centralized and decentralized.

**Table 3:** Procedures for procurement of vector control insecticides

	United Nations Regional Group							
	African		Asia-Pacific		Latin American & Caribbean		European & Others^a^	
Topic	%	(n)	%	(n)	%	(n)	%	(n)
Procurement for malaria control at central level	63	(27)	73	(30)	84	(25)	20	(10)
Procurement for arboviruses at central level	23	(26)	57	(30)	88	(25)	11	(9)
Procurement for other vector-borne diseases at central level	41	(27)	55	(29)	77	(22)	10	(10)
Procurement of vector control pesticides at decentralized level	71	(28)	72	(29)	75	(24)	50	(10)
Only WHO-recommended products procured at central level	81	(27)	87	(30)	68	(25)	29	(7)
Only WHO-recommended products procured at decentralized level	76	(21)	50	(20)	35	(17)	40	(5)
WHO quality standards used for centralized procurement	82	(28)	100	(30)	80	(25)	33	(6)
WHO quality standards used for decentralized procurement	68	(19)	52	(21)	39	(18)	40	(5)

Data presented as % of responding countries per Regional Group that gave a positive response regarding each topic (n indicates number of responding countries for each topic)

^a^ Western & Eastern Europe, North America, Australia and New Zealand

**Table 4:** Agencies other than the national-level health ministry that procured vector control insecticides

	United Nations Regional Group			
Agency	African (*n* = 19)	Asia-Pacific (*n* = 21)	Latin American & Caribbean (*n* = 18)	European & Others^a^ (*n* = 5)
Local authorities	26	48	67	80
Private sector	53	33	22	20
Donor-funded projects	37	10	22	0
Ministries other than health	11	19	17	0

Data presented as % of responding countries per Regional Group (n indicates number of responding countries per Regional Group)

^a^ Western & Eastern Europe, North America, Australia and New Zealand

WHO routinely evaluates vector control products, and publishes recommendations on approved products [17]. In 81–87% of countries in the African and Asia-Pacific regions, procurement by the central-level Ministry of Health was restricted to those products that have been recommended by WHO (Table 3). However, products that were procured by other agencies at decentralized level were less commonly restricted to WHO recommendations in most regions (35–76% of countries) (Table 3).

Vector control spraying operations could adversely affect the health of spray workers, but health risks are reduced when adequate safety precautions are taken, for example, by using personal protective equipment. National guidelines or training curricula for safety precautions or risk reduction of spray workers for vector control operations were reportedly available in 70–71% of countries in the African, Asia-Pacific and Latin American & Caribbean regions (Table 5). However, national guidelines for health monitoring of spray workers in vector control operations (e.g. to detect signs and symptoms of pesticide poisoning) were present in only 11–44% of countries, depending on the region (Table 5), suggesting a major deficiency in health monitoring. Out of a total of 28 countries with guidelines on health monitoring, 26 also had guidelines on safety precautions in place. It remains unknown to what extent these guidelines were implemented, and who implemented them.

**Table 5:** Status of application of vector control insecticides

	United Nations Regional Group							
	African		Asia-Pacific		Latin American & Caribbean		European & Others^a^	
Topic	%	(n)	%	(n)	%	(n)	%	(n)
Guidelines for safety precautions of vector control spray workers	71	(28)	70	(30)	71	(24)	56	(9)
Guidelines for health monitoring of vector control spray workers	29	(28)	27	(30)	44	(25)	11	(9)
Delegated vector control operations adequately monitored	67	(18)	54	(13)	56	(9)	50	(6)
Vector control decision-makers trained in vector control	44	(27)	38	(29)	36	(25)	25	(8)
Pest control operators required to be licensed or certified	65	(26)	63	(30)	56	(25)	88	(8)

Data presented as % of responding countries per Regional Group that gave a positive response regarding each topic (n indicates number of responding countries for each topic)

^a^ Western & Eastern Europe, North America, Australia and New Zealand

In countries where vector control operations were delegated or contracted to the private sector or to nongovernmental organizations (NGOs), these operations were monitored by the Ministry of Health in only 50–67% of the countries, suggesting that there were many delegated or contracted vector control operations that were not monitored by the health authorities (Table 5).

Furthermore, it was reported that those responsible for decision-making and implementation of vector control activities received certified training in vector control in only 25–44% of countries, which indicates a deficiency in capacity building (Table 5).

Pest control operators (PCOs) are private sector companies engaged in the control of domestic and peri-domestic pest problems, including insect pests. In 56–88% of countries across regions, PCOs were required to be licensed or certified (Table 5); licensing may or may not have involved specific training for PCO staff.

Vector control operations in which insecticides are used depend on a functional infrastructure for safe and secure transport and storage of insecticides and equipment. However, adequate, safe and secure facilities for storing vector control insecticides at periphery level were available in only 24–67% of the countries and were least common in the Latin American & Caribbean Region (Table 6). Moreover, stock keepers at periphery level with adequate training on stock management were lacking from 33 to 50% of countries across regions (Table 6). In a 33–41% minority of countries across regions it was required that the transport of vector control insecticides to stores or points-of-use was accompanied by a person trained in safe transport and emergency procedures (Table 6).

**Table 6:** Status of storage, transport, and disposal of vector control insecticides

	United Nations Regional Group							
	African		Asia-Pacific		Latin American & Caribbean		European & Others^a^	
Topic	%	(n)	%	(n)	%	(n)	%	(n)
Secure pesticide storage facilities at periphery level	46	(28)	55	(29)	24	(25)	67	(9)
Trained pesticide store keepers at periphery level	60	(25)	50	(30)	65	(23)	67	(9)
Pesticide transport personnel trained on safety, emergency	41	(27)	37	(30)	40	(25)	33	(9)
Guidance on sound disposal of vector control pesticide containers	46	(28)	48	(29)	24	(25)	22	(9)
Accumulation of obsolete vector control insecticides not a problem	56	(27)	60	(30)	48	(25)	100	(10)

Data presented as % of responding countries per Regional Group that gave a positive response regarding each topic (n indicates number of responding countries for each topic)

^a^ Western & Eastern Europe, North America, Australia and New Zealand

At the end of spray operations, empty insecticide containers (e.g. tins, flasks, sachets) should be safely disposed of to avoid their reuse or refilling, and rinsate (a mixture of pesticide with water resulting from cleaning of containers) should be reused [18]. However, 52–88% of countries across regions lacked a national guidance document on the safe and environmentally sound disposal of pesticide containers (Table 6).

Pesticides become obsolete after having expired, when their contents or packaging have deteriorated, when they are no longer needed for vector control, or when they have become de-registered or banned. Accumulation of obsolete vector control insecticides was reportedly a problem in 40–52% of countries in the African, Asia-Pacific and Latin American & Caribbean regions, but not in the European & Others Region (Table 6).

At institutional level, a national vector control unit, with the responsibility for all vector control activities, was reportedly in place in 70–88% of countries across regions, except for the European & Others Region, where it was reported from only 30% of countries (Table 7).

**Table 7:** Policy and institutional aspects of vector control

	United Nations Regional Group							
	African		Asia-Pacific		Latin American & Caribbean		European & Others^a^	
Topic	%	(n)	%	(n)	%	(n)	%	(n)
National vector control unit in place	82	(28)	70	(30)	88	(25)	30	(10)
Use of Code of Conduct for public health pesticides	78	(27)	69	(29)	54	(24)	11	(9)
Records available on use of vector control insecticides	71	(28)	82	(28)	80	(25)	44	(9)

Data presented as % of responding countries per Regional Group that gave a positive response regarding each topic (n indicates number of responding countries for each topic)

^a^ Western & Eastern Europe, North America, Australia and New Zealand

The International Code of Conduct on Pesticide Management (‘Code of Conduct’) provides a framework for governments to manage pesticides throughout their lifecycle [10]. A 54–78% majority of countries reported that their Ministry of Health used, or referred to, the Code of Conduct in the management of public health pesticides. An exception was the European & Others Region where the Code of Conduct had reportedly not been used for public health pesticides in 8 out of 9 countries (Table 7).

In 18–56% of countries, the central-level Ministry of Health did not have available records on the use of vector control insecticides, suggesting that the authorities may not keep track of the amounts and types of insecticides used in the country (Table 7).

## Discussion

The vectors of major human diseases are developing resistance to available insecticides [19–22]. Capacities needed for routine monitoring of insecticide resistance have been established in part of the countries in regions with a high burden of vector-borne diseases, as indicated in this study. Recent capacity-building efforts, in terms of a number of regional and national training courses on insecticide resistance monitoring, have likely contributed to this result [23, 24]. The standardized insecticide susceptibility tests, which measure phenotypic resistance, are the most common monitoring tools and can be implemented at a relatively low cost, but also have their limitations in terms of fluctuations in results and monitoring the intensity of resistance [5, 25–27]. Another limitation of the susceptibility test is that detected resistance may not correspond with reduced efficacy of vector control interventions in the field. Biochemical and molecular techniques are instrumental for identifying the mechanism of resistance and for detecting low frequencies of resistance genes in vector populations; however, these techniques depend on sophisticated equipment. Capacity for biochemical and molecular testing was most common in the African Region, most likely in connection with recent investments into the Region by malaria control and elimination programmes [28]. Availability of these techniques for dengue vectors was probably more limited.

Despite these positive findings about capacities for monitoring of insecticide resistance, many countries are apparently still lacking the basic capacities for management of insecticide resistance. Even though it is imperative that countries generate insecticide susceptibility data for targeted insect vectors to inform their decisions on vector control products and interventions, it is a challenge in many countries to utilize these monitoring data for optimal decision-making [23]. In addition, alternative options of insecticides with different modes of action may not be registered or available at country level. Apart from insecticide susceptibility, the evidence on the effectiveness of interventions is also an important consideration in decision-making, bearing in mind that evidence on the effectiveness of vector control methods is scarce for dengue but substantial for malaria [29, 30].

Another important finding is that, even though most countries have a system of centralized procurement of insecticides for vector-borne disease programmes in place, in many countries procurement is also taking place by other agencies including decentralized authorities. Unlike malaria programmes, dengue programmes in Asia and Latin America commonly have a decentralized organizational structure characterized by a shortage of technical expertise [31]. Centralized procurement of pesticides, as for medical supplies, has advantages over decentralized procurement in terms of efficiency, control over product selection, negotiation on price and quality, quality control, and prevention of accumulation of expired stocks. A special concern is our finding that decentralized procurements in responding countries gave less consideration to WHO-recommended products as compared to centralized procurements. Moreover, decentralized procurements are less likely to incorporate costly quality control, considering that quality control was generally weak across regions. Consequently, in many countries, procurements of vector control insecticides are taking place over which the central authorities apparently have no or little control. There is concern that substandard vector control products, particularly insecticide-treated net products, are being deployed [32], which is possibly because manufacturers did not maintain product specifications after the product had been prequalified by WHO. Hence, it is critical that quality control of vector control products is conducted as a procurement requirement or through post-market monitoring.

A related issue is that a considerable number of countries expressed difficulties in estimating the amounts needed to be procured, especially for emergency situations. Under-estimation could have serious implications for outbreak control. Over-estimation could contribute to the accumulation of obsolete pesticides; the environmentally sound disposal of obsolete pesticides is known to be very costly [33]. For countries expressing these difficulties, guidance or training tailored to their specific needs would be helpful.

Aspects of worker safety, pesticide storage practices, and pesticide waste disposal were a common weakness in vector control programmes across regions that can result in external costs of pesticides to health and the environment. This suggests that budgetary decisions by government agencies or donors have commonly emphasized the operations to achieve coverage of vector control, while financial and logistic support for health and environmental safety measures of those operations were often neglected. For example, independent observations in selected countries suggest that countries which opted for space spraying operations to control dengue in many cases did not have the available resources allocated to support health checks of spray teams, to provide insurance or compensation in case of pesticide poisoning, or even to provide the basic personal protective equipment [34]. This calls for critical review at country level. To guide policy reform, coordinated investigation into the prevalence of signs and symptoms of pesticide poisoning among vector control spray workers is needed, including on space spraying, a method which relies on airborne insecticide formulations.

The European & Others Region has scored rather poorly in the survey, particularly in relation to the capacity for insecticide resistance monitoring, availability of guidelines, and vector control training. Most countries in this region have long been relatively free from mosquito-borne diseases, apart from introduced disease cases, and endemic leishmaniasis in the Mediterranean and Central Asia [35]. However, recent outbreaks of (re-) emerging vector-borne diseases such as dengue, chikungunya and West Nile virus, together with the spread of invasive vectors, notably *Aedes albopictus* and *Ae. aegypti*, highlight the importance for countries in this region to establish adequate capacity to tackle these challenges [36–38].

The study had several limitations. The 48% country response rate suggests that the results provided a moderate representation of the targeted countries, whereas the responding countries accounted for 77% of the total population of the targeted countries, suggesting that global representation and, hence, generalisability was reasonable. The country response rate was by far the lowest in the European & Others Region and, consequently, the results from the responding countries cannot be considered representative for that region. Language barriers, for example, with Russian-speaking countries, or unavailability of the solicited data at the national level may have curbed the country response rate. Furthermore, the focal points to which the questionnaires were addressed may not have had access to accurate information regarding all questions. Another limitation of the global questionnaire was that questions and responses lacked depth and, thus, raised additional questions. To provide more insight into the situation of vector control insecticide management, a separate study in six selected countries has addressed the causes of deficiencies, the context in which decisions have been made, and the opportunities for structural improvements [34].

Special efforts on advocacy and resource mobilization are necessary to assist countries in addressing their critical shortcomings in the management of vector control insecticides. At regional level, support could be provided for regional policy development, thematic technical support across countries, and in-depth analysis and planning in selected countries [39]. At country level, vector control programmes in which insecticides are used should make adequate budgetary allocation to insecticide resistance monitoring, pesticide quality control, pesticide procurement methods, worker safety, pesticide storage, and pesticide waste disposal.

## Conclusions

Vector control interventions continue to depend largely on the action of chemical insecticides. Results from the global assessment indicated how insecticides are managed in the practice of vector control. Capacity for insecticide resistance monitoring has been established in part of the countries, often with external support; however, this capacity is still lacking from other countries. The procurement of vector control insecticides is often taking place at decentralized levels, over which the central authorities lack control, for example, on product selection or quality control. Moreover, some countries experience problems with estimating the correct amounts for procurement, especially for emergency purposes. Countries across regions showed critical shortcomings in worker safety, pesticide storage practices and pesticide waste disposal. These shortcomings call for increased attention to pesticide management in international support, budget allocation and regional collaboration (Table 8).

**Table 8:** Recommendations to countries and funding agencies, as appropriate

	Recommendation
1	Invest in capacity-building for monitoring and management of insecticide resistance
2	Establish a centralized procedure for the procurement of vector control insecticides, with provisions for pesticide quality control
3	Establish procedures and mechanisms to protect and monitor the health of vector control spray workers
4	Improve pesticide storage practices, stock management, and sound disposal of obsolete pesticides and waste

## Supplementary Information

**Additional file 1.** Topics of the analysis with corresponding survey questions

**Additional file 2.** STROBE Statement

## Data Availability

The country-anonymized dataset is available from the corresponding author upon reasonable request.

## Abbreviations

CDC: United States Centers for Disease Control and Prevention
GVRC: Global vector control response
IVM: Integrated vector management
NGO: Nongovernmental organization
PCO: Pest control operator
WHO: World Health Organization

## References

[1] Global vector control response 2017-2030. Geneva: World Health Organization; 2017.

[2] Wilson AL, Courtenay O, Kelly-Hope LA, Scott TW, Takken W, Torr SJ, Lindsay SW (2020). The importance of vector control for the control and elimination of vector-borne diseases. PLoS Negl Trop Dis.

[3] Golding N, Wilson AL, Moyes CL, Cano J, Pigott DM, Velayudhan R, Brooker SJ, Smith DL, Hay SI, Lindsay SW (2015). Integrating vector control across diseases. BMC Med.

[4] Townson H, Nathan MB, Zaim M, Guillet P, Manga L, Bos R, Kindhauser M Exploiting the potential of vector control for disease prevention. Bull World Health Organ 2005;83(12):942–947, DOI: /S0042-96862005001200017. PMC2626501 16462987

[5] Hemingway J (2014). The role of vector control in stopping the transmission of malaria: threats and opportunities. Phil Trans R Soc B.

[6] Roiz D, Wilson AL, Scott TW, Fonseca DM, Jourdain F, Müller P, Velayudhan R, Corbel V (2018). Integrated *Aedes* management for the control of Aedes-borne diseases. PLoS Negl Trop Dis.

[7] Bhatt S, Weiss DJ, Cameron E, Bisanzio D, Mappin B, Dalrymple U, Battle KE, Moyes CL, Henry A, Eckhoff PA, Wenger EA, Briët O, Penny MA, Smith TA, Bennett A, Yukich J, Eisele TP, Griffin JT, Fergus CA, Lynch M, Lindgren F, Cohen JM, Murray CLJ, Smith DL, Hay SI, Cibulskis RE, Gething PW (2015). The effect of malaria control on *Plasmodium falciparum* in Africa between 2000 and 2015. Nature..

[8] Handbook for integrated vector management. Geneva: World Health Organization; 2012. http://whqlibdoc.who.int/publications/2012/9789241502801_eng.pdf.

[9] A toolkit for integrated vector management in sub-Saharan Africa. Geneva: World Health Organization; 2016.

[10] International Code of Conduct on Pesticide Management. Rome and Geneva: Food and Agriculture Organization and World Health Organization; 2014. http://www.fao.org/fileadmin/templates/agphome/documents/Pests_Pesticides/Code/CODE_2014Sep_ENG.pdf.

[11] Global plan for insecticide resistance management in malaria vectors. Geneva: World Health Organization; 2012. http://whqlibdoc.who.int/publications/2012/9789241564472_eng.pdf.

[12] Cohen JM, Smith DL, Cotter C, Ward A, Yamey G, Sabot OJ, Moonen B (2012). Malaria resurgence: a systematic review and assessment of its causes. Malar J.

[13] Global situation of pesticide management in agriculture and public health (2019). Report of a 2018 WHO-FAO survey.

[14] van den Berg H, Gu B, Grenier B, Kohlschmid E, Al-Eryani S, da Silva Bezerra HS (2020). Pesticide lifecycle management in agriculture and public health: where are the gaps?. Sci Tot Env.

[15] United Nations Regional Groups of Member States. https://www.un.org/dgacm/en/content/regional-groups

[16] World Population Prospects 2019. New York, NY: Department of Economic and Social Affairs, Population Division, United Nations; 2020.

[17] List of WHO prequalified vector control products. Geneva: World Health Organization; 2018 http://www.who.int/pq-vector-control/prequalified-lists/PQT_VC_17July2018.pdf. Accessed: 21 January 2020; 2020.

[18] Guidelines on management options for empty pesticide containers. Rome and Geneva: Food and Agriculture Organization of the United Nations and World Health Organization; 2008.

[19] Moyes CL, Vontas J, Martins AJ, Ng LC, Koou SY, Dusfour I, Raghavendra K, Pinto J, Corbel V, David JP, Weetman D (2017). Contemporary status of insecticide resistance in the major *Aedes* vectors of arboviruses infecting humans. PLoS Negl Trop Dis.

[20] Ranson H, Lissenden N (2016). Insecticide resistance in African *Anopheles* mosquitoes: a worsening situation that needs urgent action to maintain malaria control. Trends Parasitol.

[21] Dhiman RC, Yadav RS (2016). Insecticide resistance in phlebotomine sandflies in Southeast Asia with emphasis on the Indian subcontinent. Infect Dis Poverty.

[22] Gomez MB, Diotaiuti LG, Gorla DE (2016). Distribution of pyrethroid resistant populations of *Triatoma infestans* in the Southern Cone of South America. PLoS Negl Trop Dis.

[23] Mnzava AP, Knox TB, Temu EA, Trett A, Fornadel C, Hemingway J, Renshaw M (2015). Implementation of the global plan for insecticide resistance management in malaria vectors: progress, challenges and the way forward. Malar J.

[24] Corbel V, Durot C, Achee NL, Chandre F, Coulibaly MB, David J-P (2019). Second WIN International Conference on “Integrated approaches and innovative tools for combating insecticide resistance in vectors of arboviruses”, October 2018, Singapore. Parasit Vectors.

[25] Bagi J, Grisales N, Corkill R, Morgan JC, N’Falé S, Brogdon WG, Ranson H (2015). When a discriminating dose assay is not enough: measuring the intensity of insecticide resistance in malaria vectors. Malar J.

[26] Badolo A, Traore A, Jones CM, Sanou A, Flood L, Guelbeogo WM, Ranson H, Sagnon N’F (2012). Three years of insecticide resistance monitoring in *Anopheles gambiae* in Burkina Faso: resistance on the rise?. Malar J.

[27] Sternberg ED, Thomas MB (2018). Insights from agriculture for the management of insecticide resistance in disease vectors. Evol Appl.

[28] Warren AE, Wyss K, Shakarishvili G, Atun R, de Savigny D (2013). Global health initiative investments and health systems strengthening: a content analysis of global fund investments. Glob Health.

[29] Guidelines for malaria vector control. Geneva: World Health Organization; 2019. 30844152

[30] Bowman LR, Donegan S, McCall PJ (2016). Is dengue vector control deficient in effectiveness or evidence?: systematic review and meta-analysis. PLoS Negl Trop Dis.

[31] Horstick O, Runge-Ranzinger S, Nathan MB, Kroeger A (2010). Dengue vector-control services: how do they work? A systematic literature review and country case studies. Trans Royal Soc Trop Med Hyg.

[32] Karl S, Katusele M, Freeman TW, Moore SJ (2021). Quality control of long-lasting insecticidal nets: are we neglecting it?. Trends Parasitol.

[33] Loha KM, Lamoree M, Weiss JM, de Boer J (2018). Import, disposal, and health impacts of pesticides in the East Africa rift (EAR) zone: a review on management and policy analysis. Crop Prot.

[34] van den Berg H, Velayudhan R, Yadav RS. Management of insecticides for use in disease vector control: lessons from six countries in Asia and the Middle East. PLoS Negl Trop Dis. 2021;15(4):e0009358. 10.1371/journal.pntd.0009358. 10.1371/journal.pntd.0009358 PMC8115796 33930033

[35] Dujardin J-C, Campino L, Cañavate C, Dedet J-P, Gradoni L, Soteriadou K, Mazeris A, Ozbel Y, Boelaert M (2008). Spread of vector-borne diseases and neglect of leishmaniasis, Europe. Emerg Infect Dis.

[36] Schaffner F, Medlock JM, van Bortel W (2013). Public health significance of invasive mosquitoes in Europe. Clin Microbiol Infect.

[37] Rezza G. Dengue and other *Aedes*-borne viruses: a threat to Europe? Euro Surveill. 2016;21(21):30238. 10.2807/1560-7917.ES.2016.21.21.30238. 10.2807/1560-7917.ES.2016.21.21.30238 27254392

[38] Epidemiological update: West Nile virus transmission season in Europe, 2019 [https://www.ecdc.europa.eu/en/news-events/epidemiological-update-west-nile-virus-transmission-season-europe-2019].

[39] van den Berg H, Yadav RS, Zaim M (2014). Strengthening public health pesticide management in countries endemic with malaria or other major vector-borne diseases: an evaluation of three strategies. Malar J.

