# Supplementary material for: Management of insecticides for use in disease vector control: a global survey
Source: BMC Infect Dis. 2021 May 22;21:468. doi: 10.1186/s12879-021-06155-y (PMC8141140; doi:10.1186/s12879-021-06155-y)
Supplement: Supplementary file 1 — Additional file 1. Topics of the analysis with corresponding survey questions [file 12879_2021_6155_MOESM1_ESM.docx]

Additional file 1. Topics of the analysis with corresponding survey questions

| **Capacity for insecticide resistance monitoring** | |  |
| --- | --- | --- |
|  | **Topic** | **Question** |
| 1 | Insecticide susceptibility testing | Does your country have capacity for insecticide susceptibility testing (bioassays) for monitoring of insecticide resistance of disease vectors? |
| 2 | Representative sentinel sites established | Does your country have representative sentinel sites established for monitoring of insecticide resistance of disease vectors? |
| 3 | Insectaries in place for bioassays | Does your country have insectaries in place for bioassay testing for monitoring of insecticide resistance of disease vectors? |
| 4 | Molecular testing of resistance mechanism | Does your country have capacity for molecular testing to determine the resistance mechanism of disease vectors? |
| 5 | Biochemical testing of resistance mechanism | Does your country have capacity for biochemical testing to determine the resistance mechanism of disease vectors? |
|  |  |  |
| **Conditions and challenges of procurement of vector control insecticides** | | |
|  | **Topic** | **Question** |
| 6 | Insecticide susceptibility status as criterion for selection | Is the insecticide susceptibility status of vectors used as a criterion for selection of pesticides for procurement in your country? |
| 7 | Problems estimating amounts needed for emergency situations | Does your country encounter problems with estimating the appropriate amounts of vector control pesticides to be procured for emergency situations? |
| 8 | Problems estimating amounts needed for non-emergency situations | Does your country encounter problems with estimating the appropriate amounts of vector control pesticides to be procured for non-emergency (normal/routine) situations? |
| 9 | Quality control (pre- and/or post-shipment) required for procurement | Do procurements of vector control pesticide products require quality control (pre- and/or post-shipment) in your country? |
| 10 | Procurement requirements aligned with other countries | Are procedures, requirements and guidelines for pesticide procurement aligned between the national authority and any other country in the (sub-) region? |
|  |  |  |
| **Procurement procedures of vector control insecticides** | |  |
|  | **Topic** | **Question** |
| 11 | Procurement for malaria control at central level | Does the Ministry of Health procure public health pesticides for malaria control at national level in your country? |
| 12 | Procurement for arboviruses at central level | Does the Ministry of Health procure public health pesticides for arboviruses (e.g. dengue) at national level in your country? |
| 13 | Procurement for other vector-borne diseases at central level | Does the Ministry of Health procure public health pesticides for other vector-borne disease(s) at national level in your country? |
| 14 | Procurement of vector control pesticides at decentralized level | Are there any other agencies or authorities apart from the national level procuring agency indicated in the previous question that procure pesticides for vector control in your country (e.g. sub-national, local authorities; private sector)? |
| 15 | Only WHO recommended products procured at central level | Is procurement of vector control pesticide products by the Ministry of Health in your country restricted to those recommended by WHOPES? |
| 16 | Only WHO recommended products procured at decentralized level | Is procurement of vector control pesticide products by other agencies in your country restricted to those recommended by WHOPES? |
| 17 | WHO quality standards used for centralized procurement | Are WHO quality standards for public health pesticide products (i.e. WHO specification) included in procurement requirements by the Ministry of Health in your country? |
| 18 | WHO quality standards used for decentralized procurement | Are WHO quality standards for public health pesticide products (i.e. WHO specification) included in procurement requirements by other agencies in your country? |
|  |  |  |
| **Status of application of vector control insecticides** | |  |
|  | **Topic** | **Question** |
| 19 | Guidelines for safety precautions of vector control spray workers | Are national guidelines or training curricula available for safety precautions or risk reduction of pesticide applicators (spray workers) for vector control operations? |
| 20 | Guidelines for health monitoring of vector control spray workers | Are there any national guidelines for health monitoring of pesticide applicators in vector control operations in your country? |
| 21 | Delegated vector control operations adequately monitored | If applicable, are those vector control operations which are contracted (delegated) to the private/NGO sector regularly monitored by the Ministry of Health? |
| 22 | Vector control decision-makers trained in vector control | Have those responsible for decision-making and implementation of vector control activities received certified training in vector control? |
| 23 | Pest control operators required to be licensed or certified | Does your country require pest control operators (PCOs) to be licensed or certified? |
|  |  |  |
| **Status of storage, transport, and disposal of vector control insecticides** | | |
|  | **Topic** | **Question** |
| 24 | Secure pesticide storage facilities at periphery level | Are adequate, safe, and secure facilities available for storage of vector control pesticides at periphery level in the regions? |
| 25 | Trained pesticide storekeepers at periphery level | Are keepers of vector control pesticide stocks at periphery level adequately trained on stock management? |
| 26 | Pesticide transport personnel trained on safety, emergency | Whenever vector control pesticides are transported to stores or points-of-use, is there a requirement in your country that the transport is accompanied by someone (e.g. driver, other staff) who is trained on safe transport and emergency procedures? |
| 27 | Guidance on sound disposal of vector control pesticide containers | Does a national guidance document exist on the safe and environmentally sound disposal of vector control pesticide containers (empty or used containers or sachets)? |
| 28 | Accumulation of obsolete vector control insecticides not a problem | Is the accumulation of obsolete pesticides for vector control an issue in your country? |
|  |  |  |
| **Policy and institutional aspects of vector control** | |  |
|  | **Topic** | **Question** |
| 29 | National vector control unit in place | Is there a national vector control unit/body with the responsibility for all vector control activities in your country? |
| 30 | Use of Code of Conduct for public health pesticides | Does the Ministry of Health in your country use or make reference to the International Code of Conduct on Pesticide Management in the management of public health pesticides? |
| 31 | Records available on use of vector control insecticides | Are records on the use of vector control pesticides in your country available to the Ministry of Health at the national (central) level? |
