# Supplementary material for: Management of insecticides for use in disease vector control: a global survey
Source: BMC Infect Dis. 2021 May 22;21:468. doi: 10.1186/s12879-021-06155-y (PMC8141140; doi:10.1186/s12879-021-06155-y)
Supplement: Supplementary file 2 — Additional file 2. STROBE Statement [file 12879_2021_6155_MOESM2_ESM.doc]

**Additional file 2. STROBE Statement**

Checklist of items that should be included in reports of cross-sectional studies.

|  | Item No | Recommendation |
| --- | --- | --- |
| **Title and abstract** | 1 | (*a*) The title includes the phrase “a global survey” |
|  |  | (*b*) The methods of the survey and analysis of results have been summarized in the abstract |
| Introduction | | |
| Background/rationale | 2 | The scientific background and rationale for the investigation have been reported |
| Objectives | 3 | The objective has been stated |
| Methods | | |
| Study design | 4 | Key elements of study design are present early in the paper |
| Setting | 5 | The global scale of the study, with dates, and methods of data collection have been stated |
| Participants | 6 | (*a*) all 194 Member States of WHO were selected for the survey |
| Variables | 7 | The survey questions have been included |
| Data sources/ measurement | 8* | The single source of all variables was the survey questionnaire. Data were binary responses (absence/presence). Countries were grouped by region. The absence/presence frequency of each variable among countries was compared between United Nations Regions |
| Bias | 9 | The quality of country responses depended on access to data and accuracy of reporting by respondents. This is mentioned as a limitation of the study |
| Study size | 10 | 94 countries responded out of 194 countries targeted |
| Quantitative variables | 11 | Explain how quantitative variables were handled in the analyses. If applicable, describe which groupings were chosen and why |
| Statistical methods | 12 | (*a*) No statistical methods were used |
|  |  | (*b*) Summary statistics were used to compare the results between regions |
|  |  | (*c*) Missing data were addressed in the presentation of results for each variable |
|  |  | (*d*) No analytical methods were used to take account of sampling strategy |
|  |  | (*e*) No sensitivity analysis was conducted |
| Results | | |
| Participants | 13* | (a) 194 countries were targeted, out of which 94 responded. All 94 responding countries were included in the study |
|  |  | (b) Non-participation may have occurred due to communication problems, unwillingness to participate, or unavailability of requested information |
|  |  | (c) n/a |
| Descriptive data | 14* | (a) participants represented countries |
|  |  | (b) the number of responses for each variable per region are indicated in the tables. The total number of participants per region is stated |
| Outcome data | 15* | All measures are presented in the tables |
| Main results | 16 | (*a*) Unadjusted estimates not applicable |
|  |  | (*b*) Continuous variables not applicable |
|  |  | (*c*) Estimates of relative risk not applicable |
| Other analyses | 17 | Other analyses not applicable |
| Discussion | | |
| Key results | 18 | Key results have been summarized with reference to study objectives |
| Limitations | 19 | Limitations of the study have been discussed |
| Interpretation | 20 | Overall interpretation of results in the wider context is given |
| Generalisability | 21 | The generalisability of the study results has been discussed |
| Other information | | |
| Funding | 22 | Funding information is provided |

*Give information separately for exposed and unexposed groups.

**Note:** An Explanation and Elaboration article discusses each checklist item and gives methodological background and published examples of transparent reporting. The STROBE checklist is best used in conjunction with this article (freely available on the Web sites of PLoS Medicine at http://www.plosmedicine.org/, Annals of Internal Medicine at http://www.annals.org/, and Epidemiology at http://www.epidem.com/). Information on the STROBE Initiative is available at www.strobe-statement.org.
